# SPANNER: taxonomic assignment of sequences using pyramid matching of similarity profiles

**DOI:** 10.1093/bioinformatics/btt313

**Published:** 2013-06-03

**Authors:** Michael S. Porter, Robert G. Beiko

**Affiliations:** Faculty of Computer Science, Dalhousie University, 6050 University Avenue, Halifax, Nova Scotia, B3H 4R2, Canada

## Abstract

**Background:** Homology-based taxonomic assignment is impeded by differences between the unassigned read and reference database, forcing a *rank-specific* classification to the closest (and possibly incorrect) reference lineage. This assignment may be correct only to a general rank (e.g. order) and incorrect below that rank (e.g. family and genus). Algorithms like LCA avoid this by varying the predicted taxonomic rank based on matches to a set of taxonomic references. LCA and related approaches can be conservative, especially if best matches are taxonomically widespread because of events such as lateral gene transfer (LGT).

**Results:** Our extension to LCA called SPANNER (similarity profile annotater) uses the set of best homology matches (the LCA Profile) for a given sequence and compares this profile with a set of profiles inferred from taxonomic reference organisms. SPANNER provides an assignment that is less sensitive to LGT and other confounding phenomena. In a series of trials on real and artificial datasets, SPANNER outperformed LCA-style algorithms in terms of taxonomic precision and outperformed best BLAST at certain levels of taxonomic novelty in the dataset. We identify examples where LCA made an overly conservative prediction, but SPANNER produced a more precise and correct prediction.

**Conclusions:** By using profiles of homology matches to represent patterns of genomic similarity that arise because of vertical and lateral inheritance, SPANNER offers an effective compromise between taxonomic assignment based on best BLAST scores, and the conservative approach of LCA and similar approaches.

**Availability:** C++ source code and binaries are freely available at http://kiwi.cs.dal.ca/Software/SPANNER.

**Contact:** beiko@cs.dal.ca

**Supplementary information:** Supplementary data are available at *Bioinformatics* online.

## 1 INTRODUCTION

An important step in metagenomic sequencing of environmental DNA is the taxonomic assignment of sequences to predict which genes are present in which members of a microbial sample. However, unassembled DNA sequence reads from current platforms are typically short (<600 bp) and difficult to classify (Brady and Salzberg, 2009; McHardy and Rigoutsos, 2007; Parks *et al.*, 2011). Accurate supervised classification of metagenomic sequences depends on the availability of a database of reference genomes that contains close relatives of the organisms in a microbial sample. If an organism in a community has sequenced relatives only at higher taxonomic ranks, such as class, then classification to a more precise rank will be impossible, and even assignment to the correct phylum will be difficult because of the extensive variation in DNA residue composition and gene content within most phyla (Brady and Salzberg, 2009; Parks *et al.*, 2011; Perry and Beiko, 2010).

Although best BLAST (Altschul *et al.*, 1997) matching has been used to assign taxonomy in some metagenomic studies, this approach will fail if the correct genome is not present in the reference database. In such a case, BLAST will assign taxonomy that is correct only to a certain taxonomic rank (e.g. family) and incorrect below that rank (e.g. genus and species). When a sample contains a mixture of microbes with varying degrees of taxonomic novelty, it is desirable to incorporate a measure of confidence that labels some fragments with precise ranks and others with more general ranks (i.e. a *rank-flexible* classifier: Diaz *et al.*, 2009; Parks *et al.*, 2011; Patil *et al.*, 2011). To overcome the limitations of best BLAST assignment, MEGAN (Huson *et al.*, 2007) introduced the LCA (Lowest Common Ancestor) algorithm to consider the full set of BLAST matches when assigning a read to a particular taxonomic rank and group. LCA assigns reads to the lowest taxonomic rank that is shared by all homology matches within a range of bitscores defined by the value *p* (0 ≤ *P* ≤ 1). The BLAST matches with a bitscore greater than *p* × the best bitscore will be used to generate a set (which we term here an *LCA Profile*) of BLAST matches with the highest similarity. This avoids overspecific assignment of a read to a lineage in the reference database by classifying the read to a higher taxonomic level (e.g. by assigning to the rank of family instead of species, if many different species and genera have matches that fall in the interval defined by *p*), although assignment may still be too specific if this higher taxonomic level still does not encompass the taxonomy of the read.

One limitation of LCA is the potential presence of distant taxonomic matches in the BLAST LCA Profile. For example, lateral gene transfer (LGT) involves the acquisition of genes from a potentially distantly related donor organism by a recipient. At the time of transfer, donor and recipient copies of the sequence will be identical. The two gene copies can subsequently diverge, but will remain as sisters in a phylogenetic tree and appear similar in a list of BLAST results, potentially leading to the inclusion of both in an LCA Profile. The presence of even a single distantly related taxonomic group in an LCA Profile will cause the LCA algorithm to assign a very high taxonomic rank to the result, for example, an LGT event between two phyla could lead to a sequence read being assigned to the rank of domain (e.g. bacteria) or higher (e.g. ‘cellular organisms’). Also, the effect of different evolutionary rates of different genes on classification has not been explored in depth: slowly evolving genes are likely to have more hits within a given *p* threshold, and more conservative taxonomic assignments.

Modifications to the LCA algorithm have been proposed. SOrt-ITEMS (Monzoorul *et al.*, 2009) fixes *p* at 0.9. Instead of then taking the LCA of the retained matches, a second BLAST search is done using the best BLAST match as the query, and all sequences above the *p* threshold plus the original query sequence as the new reference. Only the aligned section of each sequence is used in this reciprocal search. The LCA of all BLAST matches that are better than the match to the original query is used as the assignment. CARMA3 (Gerlach and Stoye, 2011) is similar to SOrt-ITEMS in using the aligned section of each sequence in the reciprocal search, the bitscores of which define ranges at each taxonomic rank of the reciprocal query lineage. The bitscore of the original query falls into one of these ranges, and assignment is made at that rank.

Here, we describe a new rank-flexible algorithm for taxonomic assignment called SPANNER. Instead of assigning taxonomy based on a range of matches to sequences from a set of reference genomes, SPANNER considers the similarity of the overall LCA Profile of a sequence to a reference database of LCA Profiles similarly constructed from the database of reference genomes. Classification of a query DNA sequence is based on the taxonomic diversity of its best-matching LCA Profiles. The similarity of the two LCA Profiles (query and reference) is measured using the Pyramid Match Kernel (Grauman and Darrell, 2007), which compares LCA Profiles at multiple levels of granularity to generate an overall similarity score. The lowest common ancestor of all LCA Profile matches within a range of pyramid scores (denoted by *y*) is used as the assignment, with any LCA Profile match greater than the best match × *y* (0 ≤ *y* ≤ 1) included in this range. Taxonomic assignments are, therefore, based on the similarity between the homology matching pattern of a sequence and patterns of proteins from the reference database of microbial genomes. If many proteins from a particular genome have unusual patterns of taxonomic similarity because of LGT or other evolutionary or statistical phenomena, then metagenomic reads with similar affinity patterns will be assigned in a manner that is not overly conservative. Here, we validate the SPANNER approach with a test set comprising 334 microbial genomes in a manner similar to Brady and Salzberg (2009) and a metagenome sampled from a dechlorinating community of microorganisms (Cox *et al.*, 1998).

## 2 METHODS

### 2.1 Algorithm

**Fig. 1.**
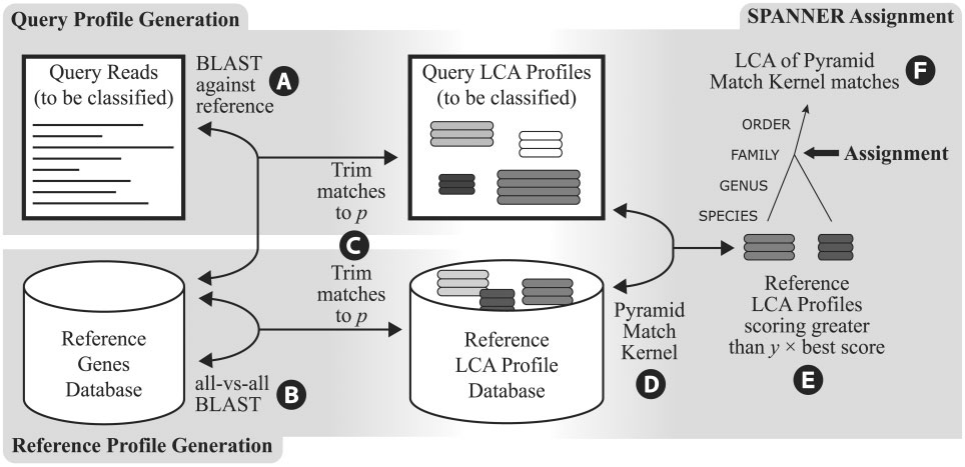
The SPANNER methodology. (**A**) All genes predicted from the query reads are compared using BLAST with the reference genes to build query LCA profiles. An LCA profile is a list of all matches, sorted by bitscore in decreasing order. (**B**) All genes in the reference database are compared using BLAST with each other to build reference LCA profiles. (**C**) All reference and query LCA profiles are trimmed: any match with a bitscore less than the best bitscore × *p* is discarded. (**D**) The PMK measures the similarity of each query to every reference LCA profile. (**E**) Any pyramid score less than the best score × *y* is discarded. (**F**) The least common ancestor of the taxonomy of the reference LCA profiles above the threshold *y* is the assignment for that query gene

SPANNER compares the LCA Profile of a query protein to LCA profiles of proteins from reference genomes with defined taxonomy. As is done in the LCA algorithm of Huson *et al.* (2007), we generate LCA Profiles by comparing the query sequence against a reference database using BLAST; the set of BLAST matches within a given bitscore range becomes the query LCA Profile (Fig. 1A). To generate LCA Profiles whose taxonomy is known, an all-versus-all BLAST of all predicted proteins from the reference set of sequenced genomes is performed, generating a set of LCA Profiles; the size of this set is equal to the number of predicted proteins from all genomes (Fig. 1B). In this article, the matches in an LCA {Profile are 2D: each match comprises the taxon matched and the e-value for that match. The bitscore range for inclusion of BLAST matches in an LCA Profile is based on setting a proportion *p*; only proteins with bitscore ≥*p* × the highest bitscore in the LCA Profile are included in the LCA Profile (Fig. 1C). Query and reference LCA profiles are then compared using a distance measure to identify the reference profiles that best match the query.

**Fig. 2.**
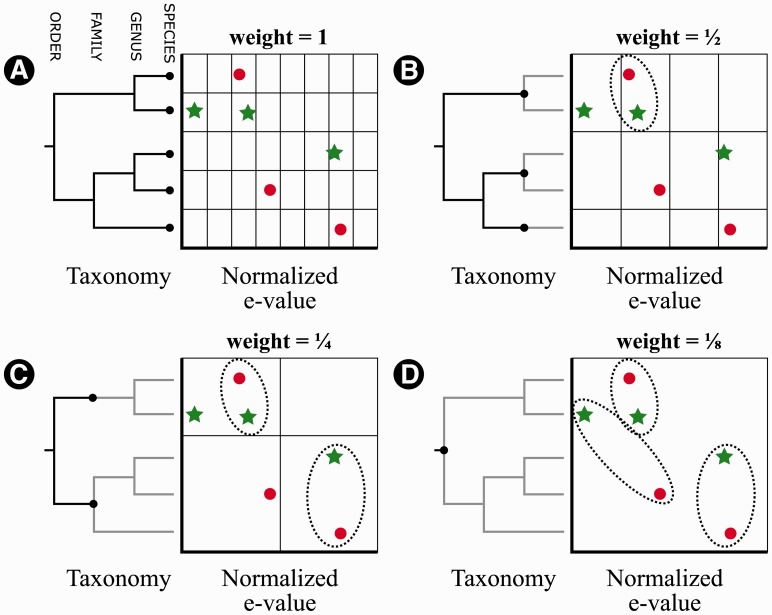
Calculating LCA profile similarity using the PMK. In this simplified example, taxonomy consists of only *h* = 4 ranks instead of the usual 8. The star profile is being compared with the circle profile, each profile consisting of three homology matches at a given species and e-value. (**A**) The initial configuration (iteration *i* = 1) of the PMK: each species in both profiles is arranged along the taxonomy axis, the hierarchy axis is divided evenly to represent these species. The e-value axis is divided into 2 *^h^* sections. The weight of an intersection at this level of granularity is 1; no intersections exist. (**B**) Iteration 2 has the e-value axis sections double in size; the taxonomy axis is divided by genus. The weight of the one intersection found is ½. The overall similarity is now ½. (**C**) Iteration 3 has the e-value axis sections again double in size, and the taxonomy axis is divided by the next higher rank of family. Two intersections are found at a weight of ¼, making the overall similarity (½) + (¼ + ¼) = 1. (**D**) By iteration *h* the e-value axis sections have doubled until only one section consists of the entire axis, likewise the taxonomy axis has reached the root of the tree (the rank of order) so that axis consists of only one section. Three intersections are counted at a weight of ⅛, making the overall similarity (½) + (¼ + ¼) + (⅛ + ⅛ + ⅛) = 1.375

Pairwise scoring of profile similarity is non-trivial because profiles will have degrees of similarity in terms of both taxonomy and match quality. For example, two profiles may both contain matches to members of the same species, genus or family, with ranges of e-value matches that are proportionately similar. An appropriate scoring scheme would assign maximum scores to pairs of profiles that are identical in both their taxonomic composition and the relative similarity of the different taxonomic hits. Weaker matches should be recognized but assigned a lower score. We adapted the Pyramid Match Kernel (PMK) (Grauman and Darrell, 2007) to calculate distances. To apply the PMK, we embed match information in a 2D grid. Genomes matched by the two profiles are placed along one axis (the *taxonomy axis*) in a manner that groups organisms by genus, family and every additional rank up to domain, and all e-values, normalized to accommodate different rates of substitution in different genes, along the other (the *e-value axis*). The e-values in each profile are normalized separately before being placed on the axis. The key property of the PMK is a hierarchical subdivision of the grid. The taxonomy axis is naturally divided into an eight-level hierarchy (*h* = 8), representing the taxonomic ranks from species to ‘cellular organisms’, whereas the continuous e-value axis is divided into 2 *h* sections. Figure 2A shows the initial configuration for the PMK, using a four-level hierarchy for simplicity. The algorithm runs in *h* iterations. At each iteration, the number of intersections between the two LCA profiles is counted and multiplied by the weight of that iteration; weights start at 1.0 for iteration 1 and are halved for each successive iteration (hence, a weight of *i*/2-1 for iteration *i*). The second iteration considers taxonomic matches at the rank of genus and subdivides the e-value axis by 2 *h-1* sections (hence, the e-value axis sections double in size); this is shown in Figure 2B. Again the intersections are counted and multiplied by a weight of 1/2. The iterations continue (Fig. 2C), increasing the size of the sections on both axes, counting the intersections and multiplying it by the weight, until at iteration *h* there is only one section spanning all of both axes (Fig. 2D). The sum of all the weighted intersection counts is the similarity between the two LCA Profiles.

Each query LCA Profile is compared with a set of reference LCA Profiles whose taxonomy is known, generating a list of matches (Fig. 1D). To preserve the rank-flexible nature of LCA, the lowest common ancestor of the top LCA profile matches is used as the final assignment. The set of best-matching profiles is generated in a manner similar to the generation of the original LCA Profiles: the best-matching reference profile is identified, and all other profiles are included whose score is greater than *y* × the score of the best-matching profile (Fig. 1E and F).

### 2.2 Validation of SPANNER algorithm

Three different types of dataset were used to assess the performance of SPANNER. All analyses were based on a set of 1210 reference bacterial and archaeal genomes acquired from NCBI in June 2010. We first applied our approach to a simulated metagenome with properties similar to those of the published ‘KB-1’ enrichment culture metagenome (Cox *et al.*, 1998). A small subset of our reference genomes was used to mimic the taxonomic novelty of KB-1, where some community members have close relatives in the reference database of sequenced genomes, whereas others are members of novel classes or phyla. Simulated metagenome sampling was also performed to mimic the distribution of contig lengths from the KB-1 metagenome. We also applied SPANNER to the real KB-1 metagenome in which the taxonomic affiliations of individual contigs is not known. Finally, we used a subset of the reference database to generate simulated reads in a taxonomic ‘leave-one-out’ framework similar to that of Brady and Salzberg (2009) and MacDonald *et al.* (2012). Although these trials do not generate entire simulated communities, they are useful to assess the performance of SPANNER at different levels of taxonomic novelty. Results obtained with SPANNER were compared with those obtained using only the LCA algorithm, assignments based on best BLAST alone and assignments using SOrt-ITEMS and CARMA3. Given the inconsistencies in species and strain naming and the challenges in assigning to the species level, all predictions were interpreted at a minimum taxonomic rank of genus.

### 2.3 Pseudometagenome

KB-1 is a dechlorinating community of microorganisms consisting mainly of *Dehalococcoides*, capable of bioremediation of tetrachlorinated ethenes into non-toxic ethene via a series of intermediates. The 16S profiling at three different time points (Duhamel and Edwards, 2006) revealed 13 different microbes in KB-1; a pseudometagenome (artificial metagenome) was created from 13 microbial genomes to model the KB-1 metagenome as closely as possible (Supplementary Table S1). For each organism found in KB-1, a proxy was chosen from a similar taxonomic group with the same degree of taxonomic novelty as the true KB-1 member. For example, a proxy for a KB-1 methanogen novel at the rank of family would be another methanogen also novel at family. The complete genome for all 13 proxies used was retrieved from NCBI. Like KB-1, the pseudometagenome included a genome novel at the phylum level (*Opitutus* from the phylum Verrucomicrobia), a mix of archaeal and bacterial genomes, four methanogens (all from the same order and three from the same class) and two members from a genus in the Spirochaetaceae family (*Treponema denticola* and *Treponema pallidum*).

A subset of the 1210 completed microbial genomes was used as the reference database for classification purposes. The 13 genomes used to build the pseudometagenome were excluded, as were genomes from the phylum Verrucomicrobia, which was necessary to make *Opitutus* unique at the phylum level when comparing it with the reference database. BLASTP version 2.2.18 was used to perform all-versus-all comparisons among reference proteins, with a maximum e-value threshold of 10^−^^3^ for inference of putative homologs. LCA profiles for the reference genomes were created by using the *P* = 0.85 bitscore threshold used by Huson *et al.* (2007). Genomic fragments from each pseudometagenome taxon were sampled in proportion to the estimated abundance of that taxon in the KB-1 culture (see Supplementary Table S1), as well as the average contig length. Proteins and protein fragments were predicted on these sampled fragments using MetaGeneMark (Zhu *et al.*, 2010) version 2.7d and compared with the reference protein set to generate query LCA Profiles. Only the highest (by bitscore) match to a genome was kept in an LCA Profile, all subsequent matches to the same genome were discarded. Query and reference LCA Profiles were compared using the PMK, with *y*, the parameter controlling the number of reference profiles included when choosing a taxonomic rank and label, set to 0.5, 0.6, 0.7, 0.8 and 0.9 in separate trials. SOrt-ITEMS and CARMA3 were also used to classify the protein sequences.

### 2.4 KB-1 metagenome

The sequences of 24 990 contigs from the KB-1 metagenome, containing 15.6 million nucleotides, were obtained from the US Department of Energy Joint Genome Institute (Sample 10 166: http://genomeportal.jgi-psf.org/aqukb/aqukb.download.html). Proteins were predicted using MetaGeneMark and then compared (using BLASTP with the same e-value threshold of 10^−^^3^) with all 1210 reference genomes to create a set of KB-1 LCA Profiles. As was done for the KB-1 pseudometagenome earlier in the text, reference proteins were compared with one another to generate reference LCA profiles. Only the highest (by bitscore) match to a given taxon was kept in any LCA Profile. The KB-1 LCA profiles were compared with the reference LCA Profiles (*P* = 0.85 and *y* = 0.95) to obtain rank-flexible assignments. The KB-1-predicted proteins were classified using LCA at a bitscore threshold of *P* = 0.85, as well as using best BLAST matching, SOrt-ITEMS, and CARMA3 for comparative purposes.

### 2.5 Leave-one-out analysis

In all, 334 microbial genomes were selected from the larger set of 1210 reference genomes (selecting at least three representatives per genus). To create a query dataset, 1000 fragments of lengths 200 and 1000 bp were sampled from random locations in each of the 334 microbial genomes. Proteins were predicted from these fragments and compared with one another in the same manner as described earlier in the text to create query LCA Profiles, with secondary matches to particular taxonomic groups ignored. The reference and query LCA Profiles were compared using a range of parameter settings (*P* = 0.65, 0.75, 0.85, 0.95 and *y* = 0.65, 0.75, 0.85 and 0.95) and at three levels of taxonomic novelty: species, genus and class. We examined the accuracy of SPANNER by using a ‘leave-one-out’ strategy to classify predicted proteins at different levels of taxonomic novelty (Brady and Salzberg, 2009; MacDonald *et al.*, 2012). To use LCA profiles at a species level of novelty, all BLAST matches to the same species were removed in both query and reference LCA Profiles. Assignments were then made at the genus level, as it is impossible to assign to the correct species. For a genus level of novelty, all BLAST matches to the same genus were removed, and assignments were made at the family level. For a class level of novelty, all BLAST matches to the same class were removed, and assignments were made at the phylum level. The predicted proteins were also classified using LCA (at the same values for *p*), SOrt-ITEMS, CARMA3 and best BLAST matching at the same levels of novelty. Examples of genes with SPANNER assignments superior to best BLAST and LCA were identified, with UPGMA trees built using PHYLIP (Felsenstein, 2005).

**Fig. 3.**
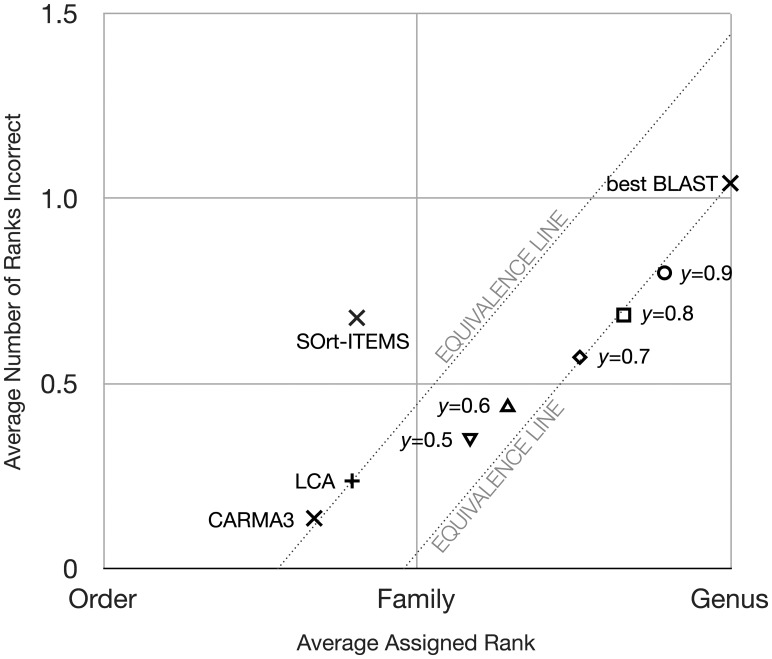
SPANNER classification of the KB-1 pseudometagenome to the genus level for a range of y-values. Diagonal lines extending from the LCA and best BLAST points connect all other points considered equivalent in terms of overall accuracy: anything on these lines introduces as many assigned ranks as incorrectly assigned ranks; therefore, any point along these lines has the same number of correctly assigned ranks. The inset shows the data relative to all taxonomic ranks, from domain (D) to species (S)

In evaluating rank-flexible predictions, we define the taxonomic *precision* (the rank at which the classification is made) and *accuracy* (the most precise rank in the prediction that is correct). For example, a given sequence may be classified to the rank of genus, but accurately only to the rank of class, in which case the classification is partially correct and partially incorrect. Our strategy for comparing predictions is illustrated in Figure 3. Taxonomic precision, shown on the *x*-axis, is the number of taxonomic ranks assigned by SPANNER or another algorithm, whether correct or incorrect. The *y*-axis shows the average number of assigned ranks that are incorrect. By treating each rank as an ordinal value from 0 (‘cellular organisms’, effectively unclassified) to 7 (species), we can compute averages over all predictions made on a given dataset. For example, an average of 3.5 ranks of taxonomic precision means that the central tendency of assignments for all proteins fell between the ranks of class and order. As accuracy is expressed as the number of ranks that are correct, we refer to two classifications as having equivalent accuracy if they have the same number of ranks correct, whether the remaining ranks are unspecified or incorrect: in Figure 3, diagonal lines show equivalent accuracy across ranges of precision and incorrect ranks. A researcher may favor an approach that either limits taxonomic errors or assigns more taxonomic ranks, and our 2D visualization makes this trade-off explicit.

## 3 RESULTS

### 3.1 The KB-1 pseudometagenome

As none of the species used to build the pseudometagenome had conspecific organisms in the reference database, correct assignments could only be made at the genus level or higher. Although BLAST matches query sequences with targets from specific strains, we limited the taxonomic precision of BLAST matches to the genus level to account for this level of taxonomic novelty; otherwise all BLAST predictions would have been guaranteed at least one incorrect rank (species). As the use of best matches yields a rank-specific classifier, all predictions were made at the genus level, even for organisms that were novel at much higher taxonomic ranks. Best BLAST matches were on average 1.041 ranks too precise (on average predictions were accurate to just below the level of family), as seen in Figure 3. LCA with *P* = 0.85 assigned proteins 1.79 ranks above genus (i.e. the ‘average’ prediction rank was between order and family), with essentially no incorrect ranks (not overspecific), as the lowest common ancestor almost always encompassed the source of the protein being assigned. LCA avoided the overspecific problem of BLASTP by assigning to higher ranks, decreasing the number of incorrect ranks at a cost of taxonomic precision. SPANNER results at different parameters (*P* = 0.85 and *y* = 0.5, 0.6, 0.7, 0.8 and 0.9) are shown in Figure 3, which on average assigned proteins between 0.21 (*y* = 0.9) and 0.83 (*y* = 0.5) ranks above genus, and between 0.79 (*y* = 0.9) and 0.35 (*y* = 0.5) ranks incorrect. SOrt-ITEMS and CARMA3 on average assigned proteins 1.17 ranks and 1.28 ranks above genus, respectively, with an average of 0.73 ranks incorrect for SOrt-ITEMS and 0.14 ranks for CARMA3. Unlike best BLAST, rank-flexible classifiers LCA and SPANNER are not guaranteed to have incorrect assignments for taxa novel at ranks higher than genus, as assignments can be made at higher ranks. SPANNER was less overspecific than best BLASTP and more precise than LCA, although only at a stringent setting of *y* = 0.9 were the SPANNER predictions slightly better overall than those of best BLAST.

**Fig. 4.**
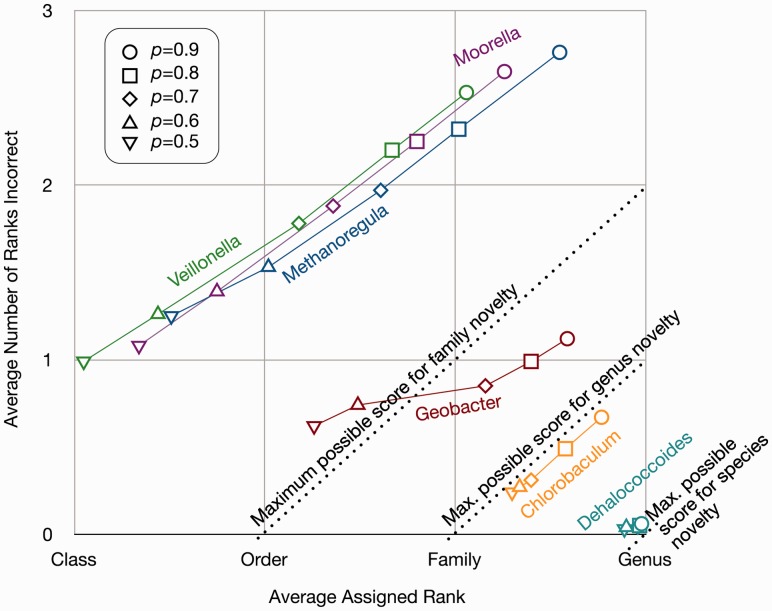
SPANNER assignments of the six most abundant taxa in the KB-1 pseudometagenome. The dotted lines represent the maximum possible score for taxa at different levels of novelty. The chosen strains of *Dehalococcoides*, *Chlorobaculum* and *Geobacter* are novel at the species level, *Moorella* and *Veillonella* are novel at the genus level and *Methanoregula* is novel at the family level

The six most abundant taxa (all taxa >4% in Supplementary Table S1) covered a wide range of taxonomic novelty with respect to the reference database and accuracy of SPANNER predictions (Fig. 4). Taxa that were only novel at low ranks (e.g. species-level novelty; having members of the same genus in the reference database) had higher accuracy than taxa novel at higher ranks. Increasing *y* improved accuracy for some taxa (e.g. *Geobacter y* ≤ 0.6 versus *y* ≥ 0.7) but not others (e.g. *Moorella* and *Veillonella* showed similar performance across all values of *y*).

### 3.2 KB-1 metagenome

**Fig. 5.**
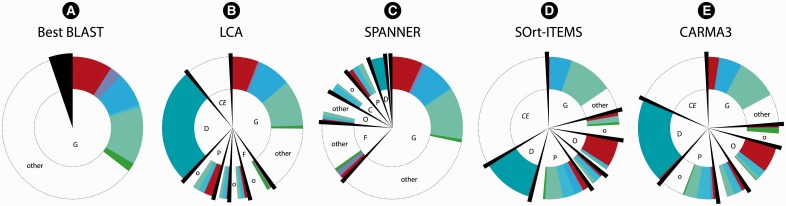
Taxonomic predictions of the KB-1 metagenome. The predicted taxonomic rank from genus (G) to domain (D) is shown (*CE*: ‘cellular organisms’), with all best BLAST assignments made at the rank of genus. The lineages of the 12 expected taxa in KB-1 are shown at the rank predicted; all other predictions are labeled ‘other’. Panels show the distribution of assigned labels, with red corresponding to Archaea, green Spirochaetes, pale green Proteobacteria, blue Chloroflexi, cyan Firmicutes and purple Chlorobi. Dark green in domain corresponds to bacteria

We next applied the five classification approaches to the 24 990 contigs assembled from the sequenced metagenome of the KB-1 dehalogenating community. Although the taxonomic affiliation of each contig is not known, the overall composition of the community has been assessed using the 16S rRNA gene as a taxonomic marker. We can, therefore, characterize global predictions in terms of expected taxonomic labels and ranks. Unlike the other classifiers, CARMA3 and SOrt-ITEMS can classify a sequence as ‘unknown’, these sequences are treated as classifications to ‘cellular organisms’ when calculating performance. In this dataset, LCA assigned proteins to a rank between class and order on average, whereas SPANNER assigned proteins to a rank between family and genus. CARMA3 and SOrt-ITEMS assigned proteins to a rank between phylum and domain on average. The taxonomic assignments of LCA, SPANNER, best BLAST, SOrt-ITEMS and CARMA3 are summarized in Figure 5, which highlights plausible assignments to the 13 identified KB-1 taxa. LCA assigned 10% of the proteins to ‘cellular organisms’, compared with 0.4% using SPANNER, 34.2% using SOrt-ITEMS (29.5% of which were classified as ‘unknown’) and 16.8% using CARMA3 (all classified as ‘unknown’). LCA, SOrt-ITEMS and CARMA3 also assigned more proteins to the rank of domain than SPANNER (28, 13 and 23% versus 5% for bacteria, assignments to Archaea was <1% for all classifiers). SPANNER assigned more proteins than LCA at the rank of class and below. Although SPANNER had greatly increased taxonomic precision relative to LCA, SPANNER had a higher proportion of assignments to taxonomic groups that are not expected to be present in the KB-1 metagenome (labeled as ‘other’ in Fig. 5). The majority of these assignments, however, were ‘nearly’ correct as the next-highest rank was often correct (Supplementary Fig. S1). At the genus level, ∼37% of LCA assignments were to genera not known to be present in KB-1, whereas the corresponding number was ∼54% for SPANNER and 63% for BLAST. SOrt-ITEMS and CARMA3 had the fewest assignments to genus (21 and 24%), but the fewest assignments to genus of Archaea present in KB-1 compared with the other classifiers. We also considered the lowest *correct* rank for each prediction made by the five algorithms (Supplementary Fig. S2).

To assess the accuracy on the metagenome, a reduced reference database of only the closest proxies to the 13 KB-1 taxa was created. For example, at least one strain of *Dehalococcoides* is present in KB-1; therefore, all five *Dehalococcoides* genomes from the original reference database were included in this reduced reference. This database contained 43 closest proxies to KB-1. Best BLAST results of KB-1 contigs against the reduced database created the gold standard of results: best BLAST could only match contigs in the metagenome to one of the 13 expected taxa. Note that this differs from the normal use of best BLAST to classify a metagenome, as the reference database has been limited only to what taxa we *a priori* assume are present. We have a higher confidence in our best BLAST gold standard for these contigs if they are also long. SPANNER assignments of proteins on contigs 50 000 bp or longer matched the gold standard for 98% of the total *Dehalococcoides* ranks and 93% of non-*Dehalococcoides* ranks.

### 3.3 Leave-one-out analysis

We used 334 genomes to construct a dataset in which the accuracy of different algorithms on reference genomes could be assessed at varying levels of taxonomic novelty by deleting all other members of the same species, genus or class (Supplementary Figs S3 and S4). When results were averaged over all training genomes, best BLAST outperformed LCA at a species level of taxonomic novelty, having more incorrectly assigned ranks but not enough to offset the increased taxonomic precision. Best BLAST performed worse than LCA at a class level of novelty. SPANNER had higher accuracy than LCA at all levels of novelty, and all values of *p* and low values of *y*, and outperformed best BLAST at high values of *p* and *y*. SPANNER was always more precise than LCA and less than best BLAST, as best BLAST matches were assigned at the most precise taxonomic rank possible. The relationship seen between LCA, SPANNER and best BLAST was consistent for fragments of length 200 and 1000 nt, overall accuracies were better (1.03, 1.09 and 1.04 times better for species, genus and class levels of novelty, respectively) on 1000 bp fragments. SOrt-ITEMS performed worse relative to the other classifiers as the novelty increased. CARMA3 assignments had fewer incorrect ranks and less precision at any novelty. Classifications to ‘unknown’ by CARMA3 and SOrt-ITEMS account for their relatively low precision: at a species level of novelty only 71.3 and 82.2% of the sequences were classified, respectively. See Supplementary Information for more details.

### 3.4 Classification of confounding genes

Events such as lateral gene transfer that would confound LCA were identified in the leave-one-out results by searching for LCA Profiles (at *P* = 0.85) where the taxonomic precision of LCA was phylum or higher, whereas SPANNER assigned to the rank of family or lower (Supplementary Table S2). One example is a sodium/glutamate symporter from *Bacillus pseudofirmus* OF4 (YP_003426830.1). This example was taken from analysis at a genus level of novelty, where all other genera within the same family are excluded, and the taxonomic assignment was at the family level (as it was impossible to correctly assign to the genus level). After removing matches to its own genus from its LCA Profile and removing matches below the threshold of *P* = 0.85, the two remaining matches are to the archaeon *Methanosarcina mazei* and the bacterium *Geobacillus sp.* Y412MC10. LCA assigned a rank of ‘cellular organisms’, whereas SPANNER correctly detected the similarities between this LCA Profile and other Bacillaceae LCA Profiles, making the correct assignment of Bacillaceae at the family level. This assignment is five ranks more precise than LCA.

**Fig. 6.**
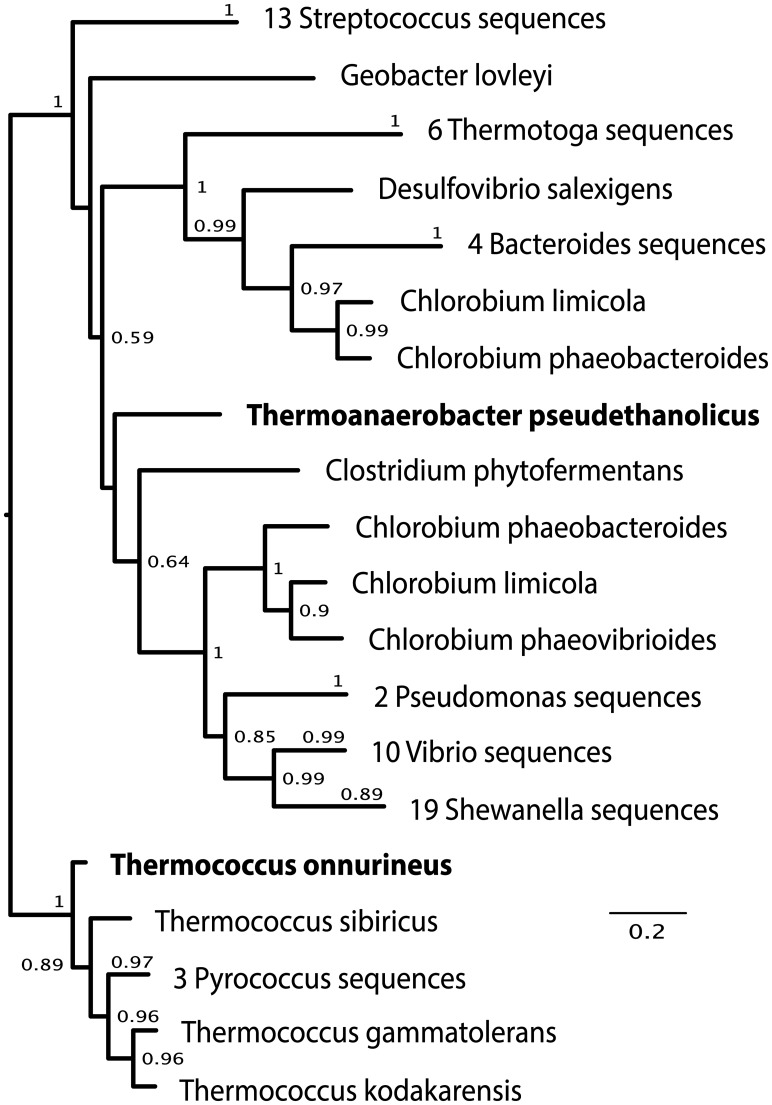
Phylogenetic tree of the LCA profile from *T.pseudethanolicus*. Although the best BLAST match was to *T.onnurineus*, the *T.pseudethanolicus* sequence is placed more closely to its taxonomic neighbors in the Clostridia

An example from the species level of novelty (assigning proteins to the genus level) is an addiction module antitoxin from *Thermoanaerobacter pseudethanolicus* (YP_001665645.1), whose LCA assignment at *P* = 0.85 is again ‘cellular organisms’ (Supplementary Fig. S5). Only one reference LCA Profile scored above the *y* threshold, from *Thermoanaerobacter sp. X514*, making a correct assignment to the genus level. This assignment is six ranks more precise than LCA. The best BLAST match for this protein is *Thermococcus onnurineus*, which is six ranks incorrect (domain to genus). SPANNER was able to match the LCA Profile from *T.pseudethanolicus* with a similar profile from *Thermoanaerobacter sp. X514*, matching the same evolutionary events in both profiles. A phylogenetic tree of the query sequence as well as the sequences in its LCA Profile are shown in Figure 6, placing the query sequence closer to its taxonomic neighbors instead of with *T.onnurineus*.

## 4 CONCLUSIONS

Supervised metagenome sequence classifiers have typically assigned reads and contigs based on similarity to reference genomes. This approach, although reasonable, does not consider the effects of variations in the rate of substitution, gene loss and gene gains from distant lineages via LGT. SPANNER exploits the situation where many genes from the same genome and from closely related genomes are expected to have similar patterns of homology matches (i.e. LCA Profiles). A gene with a broad set of BLAST matches need not generate an overly broad taxonomic prediction, if the overall similarity pattern is characteristic of the correct taxonomic group. Instead of forcing all matches to contribute to the assignment as in LCA, they can be used as features in an LCA Profile for comparison. Like the reciprocal BLAST used by SOrt-ITEMS and CARMA3, SPANNER considers the same set of matches but does not necessarily apply them to the final taxonomic assignment.

There is a trade-off between precision and incorrect ranks among the examined classifiers. SOrt-ITEMS, CARMA3 and to a lesser extent LCA, are the most conservative classifiers, with the fewest incorrectly assigned ranks but the least precision. For example, LCA assigned 25 times more sequences to the ‘cellular organisms’ level than did SPANNER on the KB-1 metagenome but had fewer assignments to ‘other’ taxa (i.e. those not expected to occur in KB-1). Using best BLAST scores in a rank-specific manner yields high taxonomic precision but many incorrectly assigned ranks. SPANNER spans the two based on the parameters *p* and *y*. The best choice of approach in a given situation might depend on the expected degree of taxonomic novelty: for example, metagenomes with many taxa novel at high ranks might be better classified with a conservative approach, such as CARMA3 or an unsupervised approach, whereas metagenomes with close proxies could be confidently assigned using SPANNER. Figure 5 shows that the five approaches assigned ‘expected’ taxonomic labels at all ranks more precise than domain in only ∼50% (SPANNER and best BLAST) and <50% (LCA, SOrt-ITEMS and CARMA3) of cases. Although this problem is partially because of the presence of taxa that are novel at high ranks, and short fragments of predicted coding sequences, it is nonetheless clear that improvements are needed if reliable predictions are to be made by any of the three approaches.

Several types of improvement to SPANNER can be envisioned. The use of a restricted target set of genomes (the ‘gold standard’ approach in Section 3.2) can drastically reduce the computational cost of SPANNER. Hybrid classifiers (Brady and Salzberg, 2009; MacDonald *et al.*, 2012) currently use the top-scoring homology assignment (e.g. best BLAST matches) in combination with compositional information. The distributional approach of SPANNER would likely decrease the number of false-positive predictions, particularly if both SPANNER and the compositional approach were used to define ‘probable sets’ from which intersecting information could be extracted. SPANNER is also complementary to other refinements of LCA such as SOrt-ITEMS (Monzoorul *et al.*, 2009) and could be combined with them.

## Supplementary Material

Supplementary Data
