# Supplementary material for: SPANNER: taxonomic assignment of sequences using pyramid matching of similarity profiles
Source: Bioinformatics. 2013 Jun 3;29(15):1858–64. doi: 10.1093/bioinformatics/btt313 (PMC3712219; doi:10.1093/bioinformatics/btt313)
Supplement: Supplementary Data [file supp_29_15_1858__index.html]

SPANNER: taxonomic assignment of sequences using pyramid matching of similarity profiles — SPANNER: taxonomic assignment of sequences using pyramid matching of similarity profiles — Supplementary Data 

# SPANNER: taxonomic assignment of sequences using pyramid matching of similarity profiles

## Supplementary Data

files

**Files in this Data Supplement:**

- Supplementary Data - docx file
- Supplementary Data - xlsx file
