# Supplementary material for: SPANNER: taxonomic assignment of sequences using pyramid matching of similarity profiles
Source: Bioinformatics. 2013 Jun 3;29(15):1858–64. doi: 10.1093/bioinformatics/btt313 (PMC3712219; doi:10.1093/bioinformatics/btt313)
Supplement: Supplementary Data [file supp_btt313_PorterBeiko_supplemental_resumbit.docx]

SPANNER: Taxonomic assignment of sequences using pyramid matching of similarity profiles

Supplemental Information

**2.3 Pseudometagenome**

**Supplemental Table S1.** The abundance of each KB-1 member in the enrichment culture (assessed using 16S sequencing), and the corresponding proxy chosen from our set of reference genomes is indicated. The novelty of each pseudometagenome proxy with respect to the reference database is given.

| Taxon in KB-1 | Abundance in KB-1 | Proxy for Pseudometagenome | Taxonomic Novelty in Pseudometagenome |
| --- | --- | --- | --- |
| *Dehalococcoides* | 56.60% | *Dehalococcoides* CBDB1 | Species |
| *Geobacter* | 7.55% | *Geobacter lovleyi* | Species |
| *Methanomethylovorans* | 1.33% | *Methanohalobium evestigatum* | Genus |
| *Methanomicrobiales* | 5.29% | *Methanoregula boonei* | Family |
| *Methanosarcina* | 1.00% | *Methanosarcina barkeri* | Species |
| *Methanosaeta* | 1.00% | *Methanosaeta thermophile* | Family |
| *Sporomusa* | 4.33% | *Veillonella parvula* DSM 2008 | Genus |
| *Acetobacterium* | 11.31% | *Moorella thermoacetica* ATCC 39073 | Genus |
| Spirochaeta SA-8 | 1.99% | *Treponema denticola* ATCC 35405 | Genus |
| Spirochaeta SA-8 2 | 1.00% | *Treponema pallidum* subsp. pallidum SS14 | Genus |
| *Syntrophus* | 1.00% | *Syntrophus aciditrophicus* | Order |
| Chlorobi SJA-28 | 4.74% | *Chlorobaculum parvum* NCIB 8327 | Species |
| OP5 | 1.00% | *Opitutus terrae* PB90-1 | Phylum |

## The KB-1 Pseudometagenome


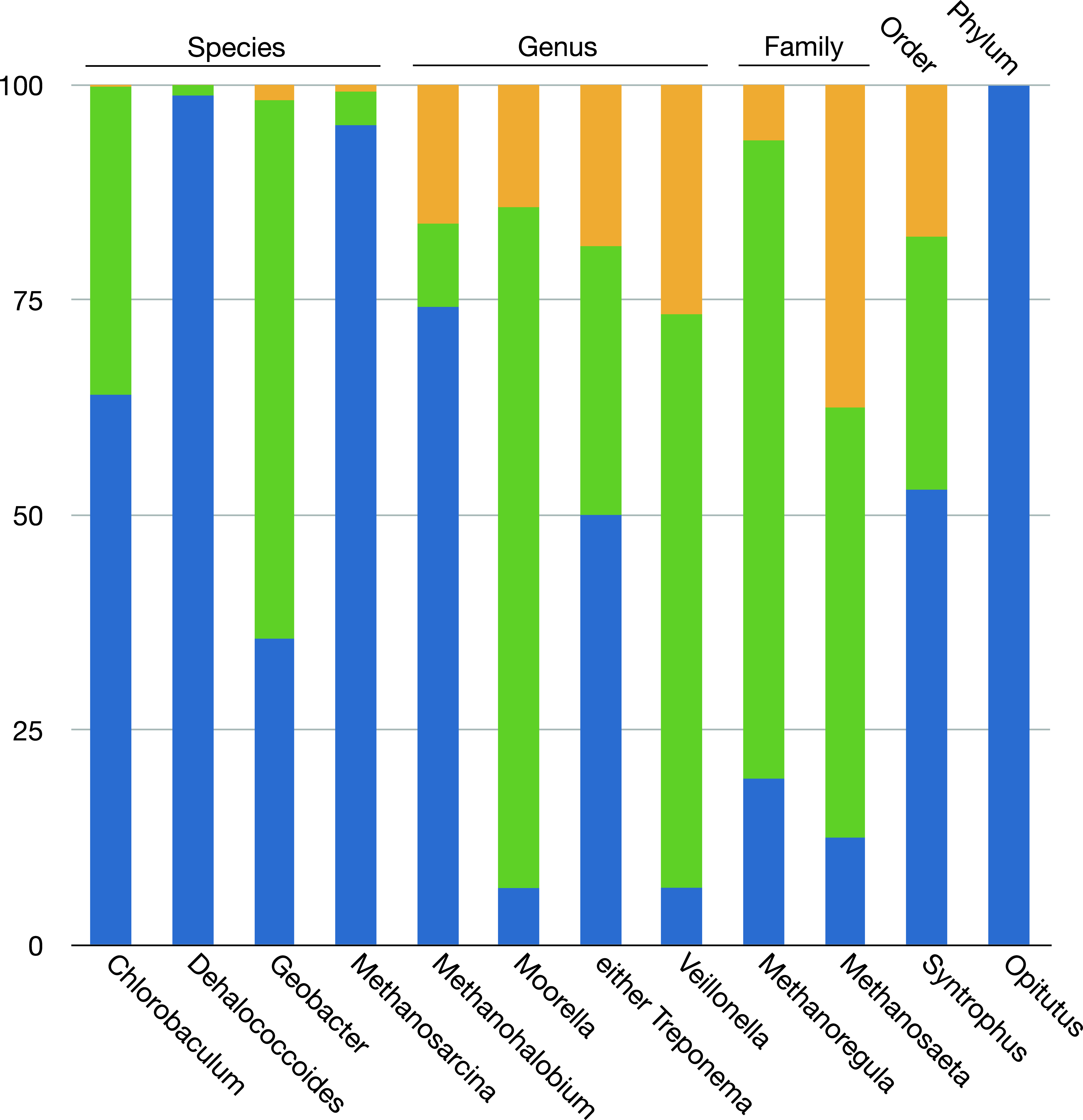


**Supplemental Figure S1.** SPANNER assignments for each taxon in the KB-1 pseudometagenome (p=0.85, y=0.9). The taxonomic novelty is for each taxon is shown at the top. All genes for each taxon are shown as the percentage of genes correctly assigned (blue), the percentage of genes correctly assigned to a higher rank (green), and the percentage of genes incorrectly assigned (orange). For taxa novel at the species level, for example, assignments correct at the rank of genus are blue; any assignment higher than genus but still correct would be green. For taxa novel to the order level, correct assignments to the rank of class are blue.

Figure S1 shows the precision of the assignment of each taxon, with pseudometagenome constituents sorted by taxonomic novelty. Taxa novel at ranks above species are more difficult to classify, with the exception of *Opitutus* (novel at the rank of phylum), which was always classified to the correct domain. Although *Geobacter* had an average of 1.1 ranks incorrectly assigned at *y*=0.9 (Figure 4), Figure 5 shows most of those incorrect ranks belonged to only 1.8% of *Geobacter* proteins, which suggests a small number of misclassifications of *Geobacter* sequences that are incorrect at high ranks such as phylum, with many of the remainder being correctly assigned to the rank of genus or family. While our test strain of *Geobacter* has a few congeners in the reference dataset, the group has few taxonomic siblings at higher ranks between genus and class. Consequently, incorrect classifications of *Geobacter* sequences (due, for instance, to absence of homologous sequences or similar profiles in reference congeners) are likely to be incorrect at even very high ranks.

## KB-1 Metagenome





**Supplemental Figure S2.** Taxonomic predictions of the KB-1 metagenome by SPANNER, LCA, best BLAST, SOrt-ITEMS, and CARMA3. The predicted taxonomic rank from genus (G) to domain (D) is shown, with all best BLAST assignments made at the rank of genus. The lineages of the 12 expected taxa in KB-1 are shown at the rank predicted; all other predictions are labelled ‘other.’ Left-hand panels (**1**) show the distribution of assigned labels and ranks for LCA (**a**), SPANNER (**b**), best BLAST (**c**), SOrt-ITEMS (**d**), and CARMA3 (**e**). Right-hand panels (**2**) show the lowest correctly assigned rank for LCA (**a**), SPANNER (**b**), best BLAST (**c**), SOrt-ITEMS (**d**), and CARMA3 (**e**).

## Leave-one-out Analysis





**Supplemental Figure S3.** Average taxonomic rank assigned for leave-one-out dataset at three different levels of taxonomic novelty. Panels show ranks assigned for leave-one-out fragments novel at the rank of species (**a**), genus (**b**) and class (**c**). As *p* increases LCA assignments approach best BLAST, likewise as *p* and *y* increases SPANNER assignments approach best BLAST. SPANNER outperformed LCA at low *y* values and outperformed best BLAST at high values of *p* and *y* at a genus level of novelty. SOrt-ITEMS performed similar to LCA at *p*=0.75 and 0.85 at a species level of novelty, with performance decreasing relative to LCA as the novelty increased. CARMA3 performed worse than the other classifiers. Results for CARMA3 at a phylum level of novelty are not shown.

Figure S3A shows analysis at a species level of novelty (where assignments are made to the rank of genus or higher). Best BLAST assigned proteins to the rank of genus and had on average 1.2 ranks incorrect for fragment lengths of 1000. LCA on the same fragments had an average taxonomic precision between class and genus, with between 0.26 and 0.82 incorrectly assigned ranks. SPANNER at *y*=0.65 had similar accuracy as LCA at the same *p*, and increased both precision and incorrect ranks as *y* increased. The most accurate SPANNER parameters were *p*=0.95 and *y*=0.95 with taxonomic precision 5.77 and 0.98 incorrect ranks, 0.01 ranks less accurate than best BLAST. SOrt-ITEMS had similar accuracy as LCA at *p*=0.85 but with 0.09 less precision and 0.12 fewer incorrect ranks. CARMA3 had the fewest incorrect ranks at 0.15, and assigned sequences between the ranks of domain and class on average. CARMA3 classified only 71% of the proteins; the remaining proteins were considered classified at “cellular organisms” which contributed to the weak classification precision. This low precision is not seen in the analysis of the KB1 pseudometagenome (Figure 3), where CARMA3 performs comparably to the other classifiers. Further investigation suggests longer sequences and an abundant taxon with low novelty (*Dehalococcoides*) in the KB1 pseudometageneome aided CARMA3 performance. Gerlach and Stoye (2011) showed that CARMA3 performs similarly or better than other classifiers suggesting its weak precision might be a characteristic of the leave-one-out dataset and not the classifier itself.

At a genus level of novelty (Figure S3B) for 1000 bp fragments, best BLAST had an average of 2.65 ranks incorrect, while LCA assigned proteins on average between phylum and family with between 0.34 and 0.98 incorrect ranks. SOrt-ITEMS assigned proteins on average at the rank of class, with 0.6 ranks incorrect. CARMA3 had the fewest incorrect ranks and lowest precision, assigning sequences between “cellular organisms” and phylum. For fragment lengths 200 bp and 1000 bp SPANNER accuracy was similar to LCA at the same *p* and *y*=0.65; as *y* increased SPANNER approached the accuracy of best BLAST. Similar trends were seen at a class level of novelty (Figure S3C).

In each trial we measured taxonomic assignments only to the level of taxonomic novelty, ignoring assigned ranks at or below this level (which are guaranteed to be incorrect). Including these ranks when measuring accuracy would add as many assigned ranks (precision; x-axis) as incorrect ranks (y-axis) causing the data points in Figure S3 to move along a diagonal equivalence line and the relative performance of the classifiers would not change. However, since rank-flexible classification can predict the novelty of a read (expressed as the rank it assigns the read to), classifiers that assign as close to the novel rank as possible are preferable to classifiers that exceed it, which our approach does not illustrate. Assignment accuracy was recalculated using any assigned rank from domain to genus regardless of novelty, measuring the extent a classifier overclassifies sequences beyond the target level of novelty (Figure S4). At a class level of novelty for sequence lengths of 1000 bp (Figure S4A1) the rank-specific classifier best BLAST made every assignment to genus according to our initial definition of BLAST classifications, increasing the average precision and average incorrect ranks by four additional ranks. CARMA3 increased by 0.01 additional ranks, SOrt-ITEMS by 0.37, LCA increased between 2.96 (*p*=0.65) and 3.66 (*p*=0.95). SPANNER increased keeping its relative position between LCA and best BLAST, suggesting it is no better at predicting a read’s novelty than LCA. LCA and SPANNER accurately predicted novelty (by making assignments close to the level of novelty but not reaching or exceeding it) at the genus level (Figure S4B) but made overly precise assignments at the class level (Figure S4A). SOrt-ITEMS accurately predicted novelty at species and class levels but made conservative assignments at the genus level. Assignments made by CARMA3 were more conservative than the novelty at all levels. Reads of 200 bp (Figure S4A2 and S4B2) were more likely to be classified to higher ranks by matching a wider set of taxa in a homology comparison, this meant on average assignments of these reads were less likely to exceed the level of novelty than reads of length 1000 bp.





**Supplemental Figure S4.** The average number of assigned ranks at or beyond the novelty of the sequence, measured to the rank of genus. The panels show a class level of novelty (**A**) where ranks are measured from class to genus, and a genus level of novelty (**B**) where only the rank of genus is measured. Sequence lengths of 1000 bp (column **1**) and 200 bp (column **2**) are shown. The panels show additional assigned ranks not counted in Figure S3.

## Improved Classification of Genes by SPANNER


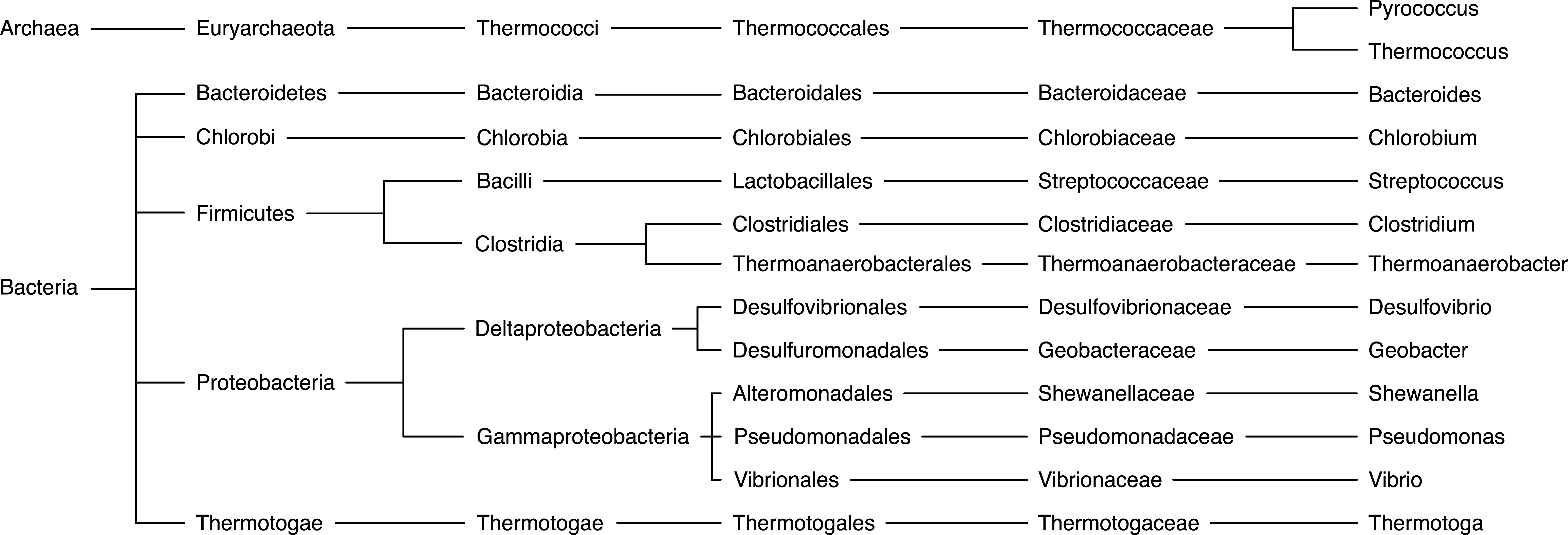


**Supplemental Figure S5.** Taxonomic tree of all genera in a *Thermoanaerobacter pseudethanolicus* LCA Profile at p=0.85 (after removing all matches to its own species). The LCA Profile contained 48 species total with e-values ranging from 10^-109^ to 10^-93^. LCA classified this as a “cellular organism” while SPANNER correctly identified the genus *Thermoanaerobacter* by matching it to a similar LCA Profile from *Thermoanaerobacter* sp. X514. The best BLAST match was to *Thermococcus onnurineus*, an archaeon (Fig. 6).

**Supplemental Table S2.** Genes from the leave-one-out trial at a genus level of novelty where LCA at *p*=0.85 assigned to more than 2 ranks higher than SPANNER. All ranks assigned by SPANNER were required to be correct for inclusion; LCA ranks were not. These genes are candidates for LGT or represent some other evolutionary process that confounded LCA.

This table is found in the file “Supplemental Table S2.xlsx”.
